# The distribution of thiamin and pyridoxine in the western tropical North Atlantic Amazon River plume

**DOI:** 10.3389/fmicb.2013.00025

**Published:** 2013-03-07

**Authors:** Laila P. Barada, Lynda Cutter, Joseph P. Montoya, Eric A. Webb, Douglas G. Capone, Sergio A. Sañudo-Wilhelmy

**Affiliations:** ^1^Marine Environmental Biology, Department of Biological Sciences, University of Southern CaliforniaLos Angeles, CA, USA; ^2^School of Biology, Georgia Institute of TechnologyAtlanta, GA, USA

**Keywords:** B-vitamin, thiamin, thiamine, pyridoxine, Atlantic, biogeochemical cycles, organic growth factors

## Abstract

B-vitamins are recognized as essential organic growth factors for many organisms, although little is known about their abundance and distribution in marine ecosystems. Despite their metabolic functions regulating important enzymatic reactions, the methodology to directly measure different B-vitamins in aquatic environments has only recently been developed. Here, we present the first direct measurements of two B-vitamins, thiamin (B_1_), and pyridoxine (B_6_), in the Amazon River plume-influenced western tropical North Atlantic (WTNA) Ocean, an area known to have high productivity, carbon (C) and dinitrogen (N_2_) fixation, and C sequestration. The vitamins B_1_ and B_6_ ranged in concentrations from undetectable to 230 and 40 pM, respectively. Significantly higher concentrations were measured in the surface plume water at some stations and variation with salinity was observed, suggesting a possible riverine influence on those B-vitamins. The influences of vitamins B_1_ and B_6_ on biogeochemical processes such as C and N_2_ fixation were investigated using a linear regression model that indicated the availability of those organic factors could affect these rates in the WTNA. In fact, significant increases in C fixation and N_2_ fixation were observed with increasing vitamin B_1_ concentrations at some low and mesohaline stations (stations 9.1 and 1; *p* value <0.017 and <0.03, respectively). N_2_ fixation was also found to have a significant positive correlation with B_1_ concentrations at station 1 (*p* value 0.029), as well as vitamin B_6_ at station 9.1 (*p* value <0.017). This work suggests that there can be a dynamic interplay between essential biogeochemical rates (C and N_2_ fixation) and B-vitamins, drawing attention to potential roles of B-vitamins in ecosystem dynamics, community structure, and global biogeochemistry.

## INTRODUCTION

The Amazon River has the largest freshwater discharge of any river into the world ocean, resulting in an influx of low-salinity, nutrient-rich water into the western tropical North Atlantic (WTNA) Ocean (Subramaniam et al., 2008). The environmental conditions resulting from the river plume, influencing approximately two million km^2^ in the WTNA, contributes to phytoplankton species succession, high rates of primary production, and a significant carbon (C) sink (Subramaniam et al., 2008). The neritic, high-nutrient areas of the plume are dominated by diatoms that utilize the nitrogen (N) and silicate (Si) supplied by the river leading to nutrient depletion in the water column. Following nutrient draw down, a shift in community composition occurs beginning with diatom-diazotroph associations (DDAs) as N becomes limited but sufficient supplies of Si are still present. This is followed by a subsequent community shift to more typical oceanic N_2_ fixing organisms such as *Trichodesmium* spp*.* (Wood, 1966; Capone et al., 1997; Carpenter et al., 1999; Foster et al., 2007). The succession of phytoplankton species supports an extensive area of increased C and dinitrogen (N_2_) fixation resulting in a C sink of approximately 1.7 Tmol annually (Subramaniam et al., 2008). Although many of the factors that limit C and N_2_ fixation in this region have been extensively studied, the roles of organic growth factors such as B-vitamins have not been investigated despite their biological importance. With recent advances in analytical methodologies that directly measure B-vitamins in marine systems (Sañudo-Wilhelmy et al., 2012), we can now start understanding the processes influencing the distribution and concentrations of B-vitamins in the world ocean. River and groundwater inputs are thought to be sources of B-vitamins as their concentrations have been inversely correlated with salinity (Gobler et al., 2007) and river plumes have previously been shown to transport macronutrients and trace metals to the ocean (Boyle et al., 1982; Tovar-Sanchez and Sañudo-Wilhelmy, 2011). However, the transport of dissolved B-vitamins from rivers to the coastal ocean has never been evaluated. This study represents the first attempt to establish the importance of the Amazon River as a source of some B-vitamins to the WTNA Ocean.

B-vitamins are essential coenzymes for many diverse biochemical reactions, including enzymes in the Calvin cycle, amino acid biosynthesis, the tricarboxylic acid cycle (TCA cycle), and nucleic acid metabolism (Voet et al., 2001). Fitting with their central role in metabolism, B-vitamins were recognized as important promoters of bacterial growth as early as the 1930s (McDaniel et al., 1939) and by the 1950s were found to be essential for the cultivation of many marine and freshwater algae (Provasoli and Pintner, 1953). Recent studies have confirmed the ecological relevance of B-vitamins in the environment by demonstrating their ability to limit or co-limit phytoplankton growth and biomass (Panzeca et al., 2006; Bertrand et al., 2007), including harmful algal blooms (Tang et al., 2010). Furthermore, Sañudo-Wilhelmy et al. (2012) recently showed that large areas of the ocean are vitamin depleted. However, no study has addressed the influence of some B-vitamins on C and N_2_ fixation in the Atlantic Ocean, and herein we describe the potential relationship between two B-vitamins, thiamin (B_1_), and pyridoxine (B_6_), concentrations and biogeochemical rates in the Amazon-influenced WTNA Ocean.

Vitamin B_1_ is an essential organic growth factor required by most organisms, and plays an integral role in biogeochemical reactions involving C transformations (Henkes et al., 2001; Jordan, 2003; Pohl, 2004). It functions by associating with a number of important enzymes including pyruvate dehydrogenase, which bridges glycolysis and the citric acid cycle, as well as transketolase, which plays a critical role in the Calvin cycle (C fixation reactions of photosynthesis) and the pentose phosphate pathway (Henkes et al., 2001; Jordan, 2003). Many bacteria and Protista have been shown to require vitamins.

Vitamin B_6_ was first identified in 1932 by Ohdake (Ohdake, 1932), and is now known to catalyze over 160 biochemical reactions that mainly involve amino acid transformations (Snell, 1953; Percudani and Peracchi, 2009). Because the role that the amino acids glutamine and glutamate have in the assimilation of ammonia (NH_3_), the product of N_2_ fixation, which is incorporated into two amino acids (Staley et al., 2007), we hypothesized that vitamin B_6_ concentrations and availability could therefore also influence the N cycle.

Previous field and laboratory studies have focused on the vitamins B_1_, B_7_, and B_12_ as they were thought to be required for growth, while other B-vitamins (e.g., B_6_) were largely ignored (Provasoli and Pintner, 1953; Droop, 1957; Burkholder and Burkholder, 1958; Carlucci and Bowes, 1970,^1972^; Strickland, 2009). This paradigm shifted when the genome of one of the most abundant bacteria in the ocean, *Pelagibacter ubique*, was first published revealing the absence of the genes required for the biosynthetic pathways of vitamins B_1_ and B_6_ (Giovannoni et al., 2005). *P. ubique* belongs to the SAR11 clade, which accounts for a third of all heterotrophic cells present in surface waters (Morris et al., 2002), and thus plays a large role in the global carbon cycle (C cycle). Subsequently, the genes required for the *de novo* synthesis of B-vitamins were found to be absent from bacteria belonging to the SAR86 clade, which are highly abundant uncultured members of marine surface bacterial populations (Dupont et al., 2011). In fact, over half of marine phytoplanktonic species investigated thus far are auxotrophic, which includes some of the most abundant and ubiquitous marine species (Croft et al., 2006), highlighting the importance of external sources of B-vitamins, including vitamin B_1_. These genomic data suggest that exogenous B-vitamin pools are essential for the survival of some marine plankton, as they rely solely on the environment to meet their B-vitamin requirements. The availability of vitamins B_1_ and B_6_ may therefore play a significant role in N and C cycling, and may be a previously unknown factors contributing to the regulation of the “biological carbon pump.” However, little is known about the sources and sinks of B-vitamins in marine systems, or how they cycle between vitamin producers and consumers.

Despite the biologically important role vitamins B_1_ and B_6_ play in ecologically relevant enzymes involved in C and N cycling, primarily carbohydrate and amino acid metabolism, little is known about their concentrations or distributions in marine systems. The objectives of this study were (1) to provide the first directly measured depth profiles of vitamins B_1_ and B_6_ in a highly productive region of the WTNA, (2) to determine the spatial distributions of those vitamins in that region, (3) to determine the influence of the Amazon River Plume on that spatial gradient, and (4) to determine the importance of these vitamins in C and N cycles.

## MATERIALS AND METHODS

**FIGURE 1:**
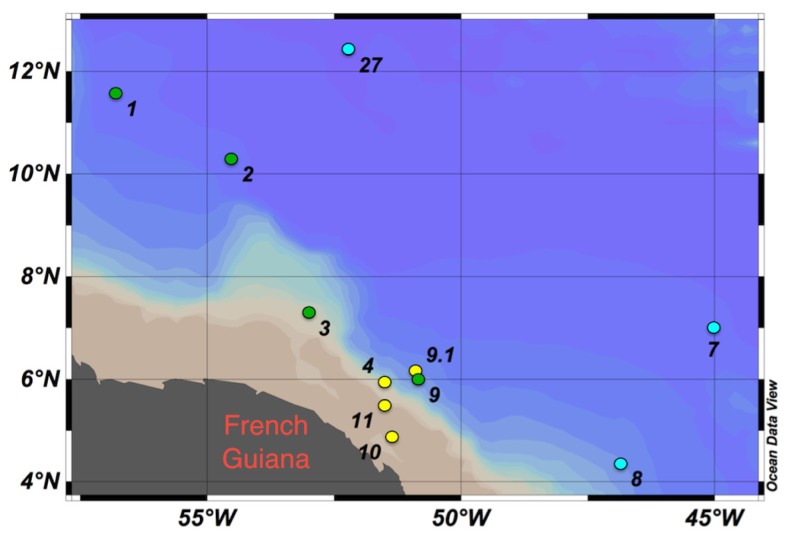
**Study sites in the western tropical North Atlantic (WTNA) Ocean with degrees latitude north and degrees longitude west shown.** Stations clustered by sea surface salinity (SSS): low-salinity stations (SSS < 30, yellow circles), mesohaline stations (30 <SSS>35, green circles), and oceanic/open ocean stations (SSS > 35, blue circles). Ocean data view (Schlitzer, 2011).

Samples were collected in the WTNA on board the R/V Knorr as part of the Amazon influence on the Atlantic: carbon export from nitrogen fixation by diatom symbioses (ANACONDAS) project from May 23 to June 22, 2010. Sampling stations were between longitude −56.8°E and −45.0°E and latitude 4.3°N and 12.4°N (**Figure 1**). Stations were grouped by sea surface salinity (SSS) and designated as low-salinity (SSS < 30, stations 4, 9.1, 10, and 11), mesohaline (30 < SSS > 35, stations 1–3, and 9), and oceanic (SSS > 35, stations 7, 8, and 27).

Vitamin samples were collected from the top 150 ms using a Niskin bottle rosette sampler and filtered through a 0.2-μm Supor filter (PALL, Life Sciences) using a peristaltic pump. The filtrate was collected in 250 ml acid cleaned high density polyethylene (HDPE) bottles and frozen until analysis. Vitamin samples were extracted and pre-concentrated according to the method of Sañudo-Wilhelmy et al. (2012). Briefly, samples were passed through solid-phase C18 resin at a flow rate of 1 mL/min to concentrate vitamins. Samples were adjusted to pH 6.5 before being passed through the resin, and then adjusted to pH 2.0 to obtain maximum vitamin recovery. Vitamins were subsequently eluted off the columns with methanol, dried, and dissolved in 200 μl of MilliQ water. Vitamin concentrations were then quantified using liquid chromatography/tandem mass spectrometry (LC/MS/MS). Each extraction included a blank and spiked positive control to test for contamination and extraction efficiency. Detection limit of vitamins B_1_ and B_6_ were 0.81 pM and 0.61 pM, respectively (Sañudo-Wilhelmy et al., 2012). Some controls used for estimating extraction efficiency with a vitamin spike were compromised by vitamin-contaminated DI water yielding in some cases efficiency greater than 100%. However, for most of the samples, extraction efficiency was close to 100%.

Chlorophyll a (Chl a) samples were collected from a Niskin bottle rosette into 1L amber bottles, filtered onto 25 mm GF/F filters and analyzed according to the EPA modified fluorometric method 445.0 (Arar and Collins, 1997) in a Turner Designs Fluorometer. Sample volumes ranged from 500 mL to 1 L depending on biomass. In general, oceanic stations utilized 1L volumes, while mesohaline and low-salinity stations had higher biomass allowing only 500 mL volumes to be filtered.

N_2_ fixation and C fixation were performed according to the method of Montoya et al. (1996) and Montoya and Voss (2006) using 4 L polycarbonate bottles completely filled and equipped with silicone rubber caps. Bottles were enriched with 3 mL of 99% ^15^N_2_ (Isotec) and 250 μL of 0.1 M NaH^13^CO_3_ (Sigma). After on-deck incubation for 24 h at surface seawater temperature and simulated conditions of light for the collection depth, bottles were pre-filtered through 10-μm Nitex mesh onto pre-combusted GF/F filters. Material on the 10-μm filter was washed onto GF/F filters. Filters were dried and stored until mass spectrometric analysis in the laboratory. Isotope abundances were measured by continuous-flow isotope ratio mass spectrometry using a CE NA2500 elemental analyzer interfaced to a Micromass Optima mass spectrometer.

Statistical analysis was performed using SigmaPlot's (Systat Software Inc.) *T*-test except when assumptions of normality and equal variance were violated resulting in the use of the non-parametric Mann–Whitney rank sum test. The degree to which C and N_2_ fixation correlated with each of the B-vitamins was evaluated by means of a Pearson product moment correlation test. Linear regression models were performed using R v2.12.2 statistical programming language (R Development Core Team, 2012). Exhaustive step-wise general linear regression models and leave one out cross validation for generalized linear models utilized the following packages: boot (Canty and Ripley, 2012), leaps (Lumley and Miller, 2009), random Forest (Liaw and Wiener, 2002), and data analysis and graphics (DAAG; Maindonald and Braun, 2012). Due to missing data, the parameters omitted from this analysis were PAR, Chl a, and cell counts.

## RESULTS

### CONCENTRATIONS OF B-VITAMINS

**FIGURE 2:**
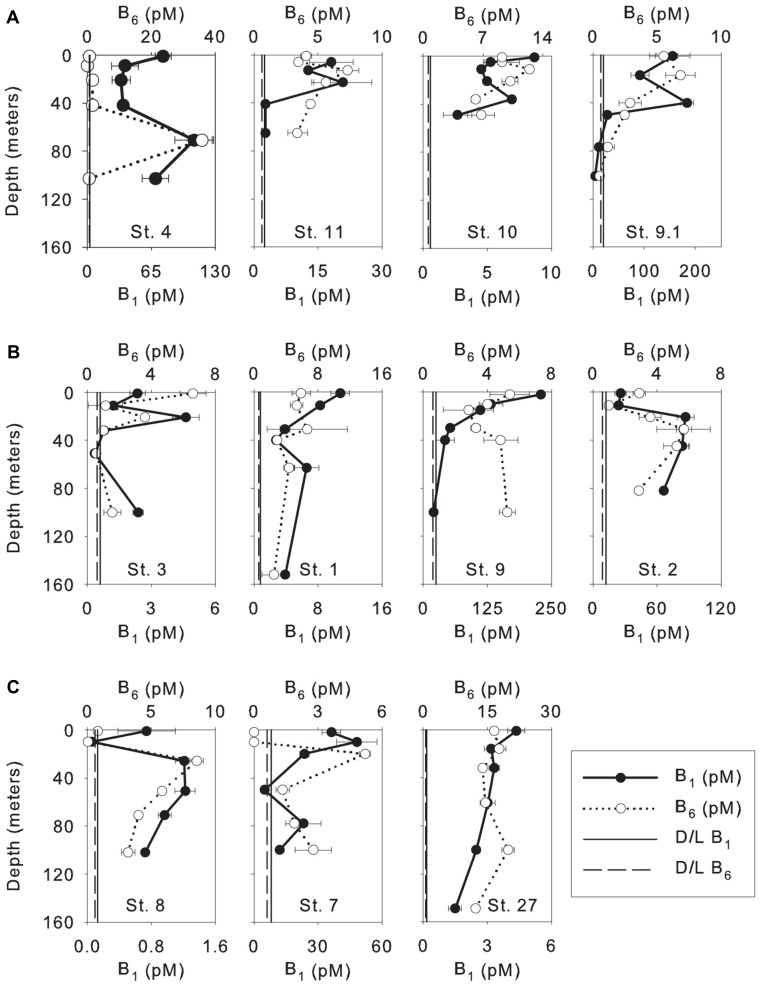
**Depth profiles of dissolved vitamins (B_1_ and B_6_) measured in the WTNA Ocean; (A) low-salinity stations; (B) mesohaline stations; (C) oceanic/open ocean stations.** Stations are ordered by sea surface salinity (SSS) moving from the lowest to highest SSS. Surface concentration of vitamin B_1_ for station 11 (964.8 ± 426 pM) omitted due to concerns with possible contamination and for visualization of variation within the depth profile (average concentrations ± 1 standard deviation). Vertical lines show the detection limit (D/L) of vitamin B_1_ (solid line) and B_6_ (dashed line).

Vitamin B_1_ in the WTNA varied widely among stations and ranged from undetectable to 229 pM (**Figure 2**), except for the surface sample at station 11 measuring 964 pM and was suspected to be compromised by sample contamination. The lowest concentrations of vitamin B_1_ were measured at the oceanic stations (undetectable to 50 pM) followed by low-salinity stations (2.5–184 pM), and the highest concentrations were observed at mesohaline stations (undetectable to 229 pM, **Figure 2**). Vitamin B_6_ concentrations also varied widely among stations ranging from undetectable to 36 pM. B_6_ concentrations were lowest at the mesohaline stations (undetectable to 7 pM) followed by oceanic stations (undetectable to 20 pM), and were highest at low-salinity stations (undetectable to 36 pM, **Figure 2**). In general, higher concentrations of B-vitamins were found at lower salinity stations and were significantly higher in the surface plume water at some stations suggesting a riverine source (**Table 1**). There was no clear spatial trend observed between the two vitamins suggesting they function and behave differently from one another, and the high variability suggests a dynamic behavior influenced by sources and sinks.

**Table 1:** Statistical test results comparing B vitamin concentrations at surface depths with below surface or halocline depths. Vitamin B_1_ followed by B_6_, the specific statistical test, failed *T*-test assumptions, and *p* value for each station is listed.

Station	Station type	Surface concentrations	Test	Failed assumption	*p* value
**B**_**1**_					
1	Mesohaline	Higher	Mann–Whitney	Normality	0.002
2	Mesohaline	Lower	Mann–Whitney	Equal variance	<0.001
7	Oceanic	Higher	*T*-test		<0.001
9	Mesohaline	Higher	*T*-test		<0.001
9.1	Low-salinity	Higher	Mann–Whitney	Normality	0.044
10	Low-salinity	Higher	*T*-test		0.018
11	Low-salinity	Higher	Mann–Whitney	Normality	0.009
27	Oceanic	Higher	*T*-test		0.006
**B**_**6**_					
2	Mesohaline	Higher	*T*-test		0.001
7	Oceanic	Lower	Mann–Whitney	Normality	0.005

### POTENTIAL EFFECT OF B-VITAMINS ON BIOLOGICAL PROCESSES

**FIGURE 3:**
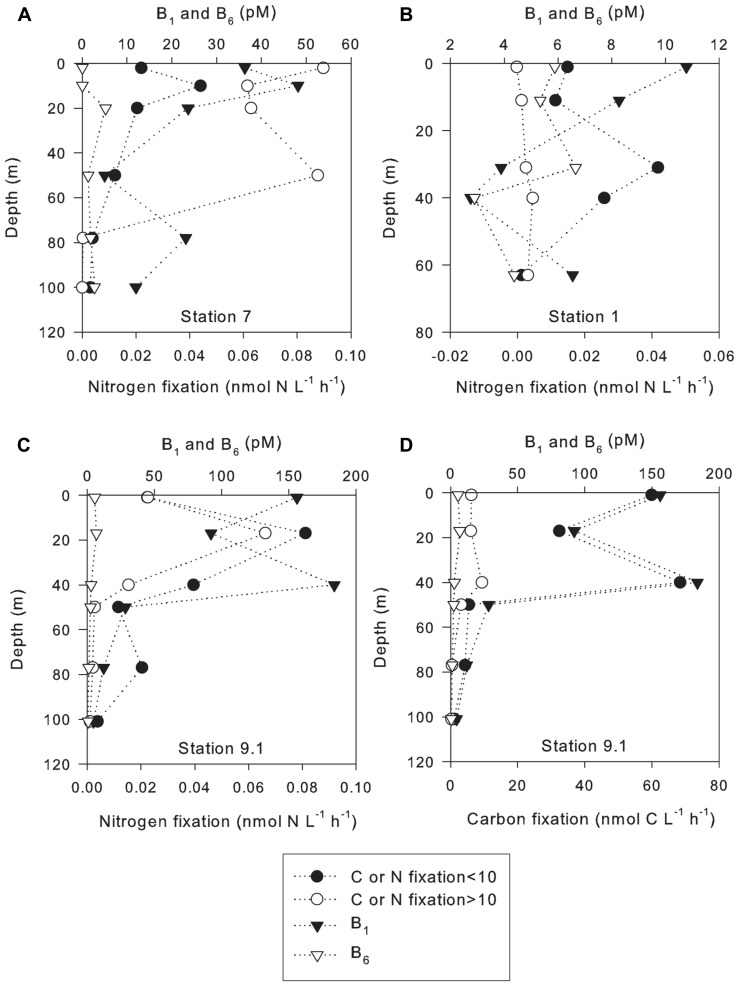
**Carbon and nitrogen fixation rates in the less than and greater than 10-μm size classes with B vitamin depth profiles. (A)** Station 7; **(B)** Station 1; **(C)** Station 9.1; **(D)** Station 9.1.

N_2_ fixation rates were positively correlated with vitamin B_1_ concentrations at station 7, 8 (in the small size class), 9.1, and 10 (**Table 2**). N_2_ fixation rates were inversely correlated with vitamin B_1_ at stations 1, 4, and 8 (in the large size fraction, **Table 2**). N_2_ fixation rates were positively correlated to vitamin B_6_ concentrations at station 1 (in the small size fraction), 8, 9.1, and 10 (in the large size fraction, **Table 2**). N_2_ fixation rates were negatively correlated to vitamin B_6_ at stations 1 (in the large size fraction), 4, 7, and 10 (in the small size fraction, **Table 2**). However, significant relationships between increases in N_2_ fixation rates and vitamin B_1_ concentrations were only observed at station 7 in the small size class (*p *value 0.045, **Figure 3A**). A significant inverse relationship was observed at station 1 in the large size fraction (*p* value 0.029, **Figure 3B**). Significant relationships between increases in N_2_ fixation rates and vitamin B_6_ concentrations were only observed at station 9.1 in the large size class (*p* value 0.017, **Figure 3C**).

**Table 2:** Correlation coefficients of vitamins B_**1**_ and B_**6**_ with nitrogen and carbon fixation in the less than and greater than 10-μm size classes, direction of relationships, correlation coefficients, and *p* values.

Correlation coefficients (*p* value)					
Station	*N* fix <10	*N* fix >10	*C* fix <10	*C* fix >10
**B**_**1**_				
1	−0.581 (0.304)	−0.916 (0.029)	0.236 (0.703)	−0.694 (0.194)
4	−0.072 (0.86)	−0.358 (0.35)	0.183 (0.64)	0.073 (0.85)
7	0.820 (0.045)	0.215 (0.68)	0.597 (0.21)	0.103 (0.85)
8	0.288 (0.580)	−0.189 (0.760)	−0.174 (0.742)	−0.416 (0.413)
9.1	0.430 (0.40)	0.395 (0.44)	0.998 (<0.001)	0.95 (0.004)
10	0.691 (0.129)	0.547 (0.261)	0.430 (0.394)	0.609 (0.200)
**B**_**6**_					
1	0.315 (0.606)	−0.697 (0.191)	0.659 (0.227)	0.177 (0.776)
4	−0.200 (0.61)	−0.273 (0.48)	−0.416 (0.27)	−0.290 (0.449)
7	−0.392 (0.44)	−0.301 (0.56)	−0.394 (0.44)	−0.024 (0.964)
8	0.627 (0.183)	0.307 (0.616)	−0.0728 (0.891)	−0.0547 (0.918)
9.1	0.780 (0.067)	0.892 (0.017)	0.58 (0.227)	0.645 (0.167)
10	−0.0741 (0.889)	0.133 (0.801)	0.190 (0.718)	0.195 (0.711)

Carbon fixation rates were positively correlated with vitamin B_1_ at stations 1 (in the small size fraction), 4, 7, 9.1, and 10 (**Table 2**). Carbon fixation rates were inversely correlated to vitamin B_1_ at stations 1 (in the large size class) and 8 (**Table 2**). Carbon fixation rates were positively correlated with vitamin B_6_ at stations 1, 9.1, and 10 (**Table 2**). Carbon fixation rates were inversely correlated with vitamin B_6_ at stations 4, 7, and 8 (**Table 2**). However, significant increases in C fixation rates with increasing B_1_ concentrations were only observed at station 9.1 in both size classes (*p* values 0.000008 and 0.004, respectively, **Figure 3D**). No significant relationships between vitamin B_6_ concentrations and rates of C fixation were observed.

### LINEAR REGRESSION MODELS

**FIGURE 4:**
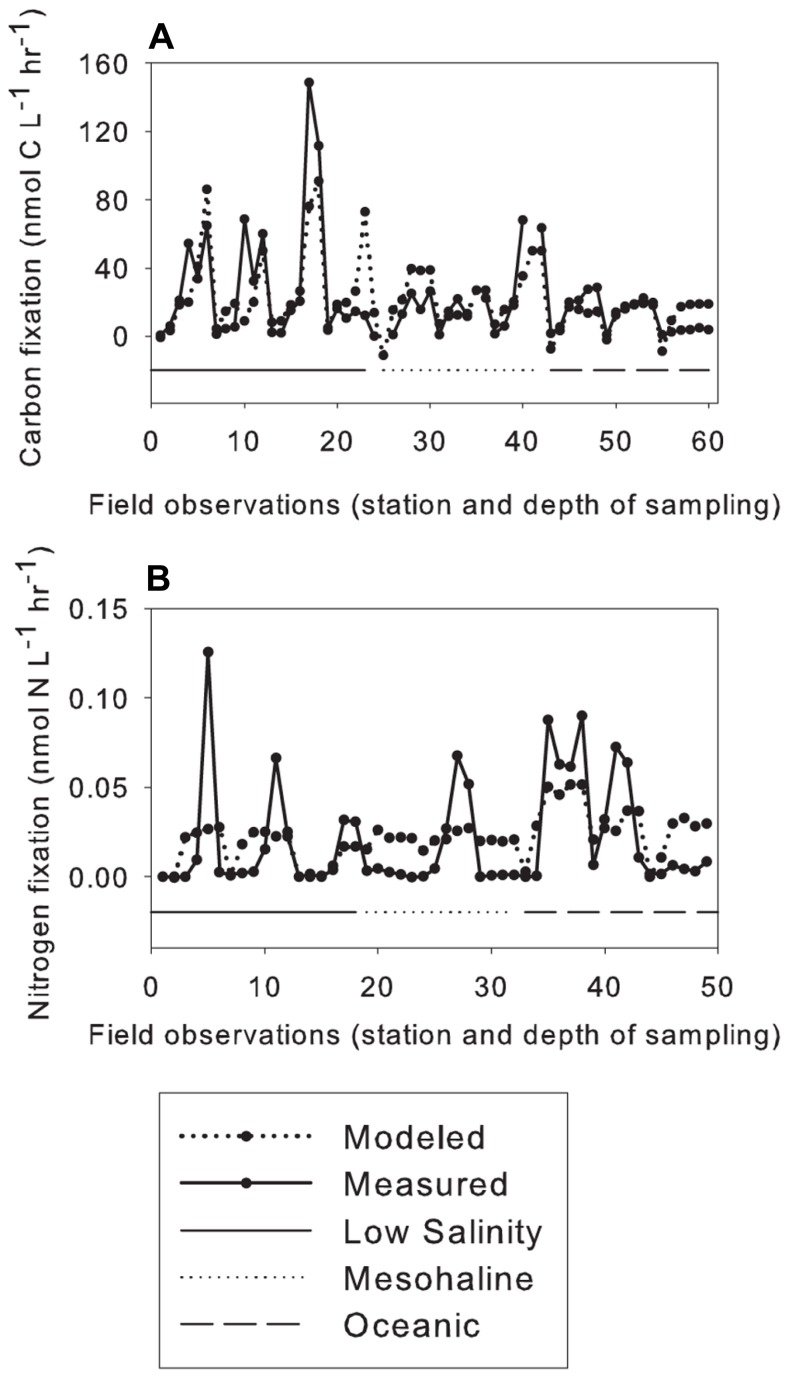
**Multiple linear regression models.** Nutrients; phosphate, PO_4_^3-^; silicate, dSi; thiamin, B_1_; mixed layer depth, MLD; photosynthetically active radiation, PAR; fluorescence, fluor.; and temperature (°C), Temp. Stations are separated by sea surface salinity (SSS), solid line below low-salinity stations, dotted line below mesohaline stations, and dashed line below oceanic stations, **(A)** Carbon fixation <10-μm size class, and (**B**) Nitrogen fixation >10-μm size class.

Linear regression models included data from all stations except for station 9 where N_2_ and C fixation data were not available. Tests were performed omitting Chl a, photosynthetically active radiation (PAR), and/or cell counts due to missing data. The linear model showed that the factors correlating with C fixation in the small size class included Si, vitamin B_1_, and water temperature (**Figure 4**). The model was significant with a *p* value of 8.83 × 10^−11^, predictive error (the average deviation between the known values and the models predicted values) of 721, and an *R*^2^ value of 0.522 (**Table 3**). The model predicting N_2_ fixation in the larger size class showed the most important factors were temperature, mixed layer depth (MLD), and vitamin B_6_ (**Figure 4**). The model was significant with a *p* value of 3.92 × 10^−4^, predictive error of 6.7 × 10^−4^, and an *R*^2^ value of 0.241 (**Table 3**).

**Table 3:** Multiple linear regression model factor coefficients and statistical results for carbon fixation in the less than 10-μm size class and nitrogen fixation in the greater than 10-μm size class.

Model	Factor coefficients	Intercept	Predictive error	Adjusted *R*-squared	*p* value
**C**							
	Si	B_1_	Temp				
	1.81E+00	−7.43E-02	4.25E+00	−1.04E +02	7.21E +02	5.22E-01	8.83E-11
**N**							
	Temp	MLD	B_6_				
	7.03E-03	4.72E-04	−1.21E-03	−1.80E-01	6.70E-04	2.41E-01	3.92E-04

## DISCUSSION

This is the first study to measure directly the B-vitamins, B_1_ and B_6_, in the WTNA euphotic zone within the influence of the Amazon River plume. We observed high variability in the concentrations and distributions of these vitamins in the area of study. Vitamin B_1_ was found below the limit of detection at mesohaline station 3 (31 and 51 m) and oceanic station 8 (2, 10, and 100 m), and vitamin B_6_ was found to be below the limit of detection at low-salinity station 4 (8 m), mesohaline stations 3 and 9 (51 and 100 m, respectively, and oceanic station 8 (10 m). The low concentrations of B-vitamins and high spatial variability observed were consistent with previous studies. In fact, in large regions of the Eastern Pacific Ocean between 24°N and 34°N, B-vitamins were found to be below the limit of detection (Sañudo-Wilhelmy et al., 2012). The ranges of vitamin B_1_ concentrations measured in this study (0.05 to ∼1000 pM) are consistent with previously published results from both bioassays and direct measurements (**Table 4**). The concentration of B_1_ measured using bioassays ranged from 33 to 1633 pM in the North Pacific Ocean (Natarajan and Dugdale, 1966), 36 to 1500 pM in the subarctic Pacific Ocean (Natarajan, 1970), 16 to 133 pM in the Southern California Coastal zone (Eppley et al., 1972), and direct measurements of vitamin B_1_ ranged from 200 to 600 pM in the Stony Brook Harbor channel and Peconic River (Okabamichael and Sañudo-Wilhelmy, 2005), 0.7 to 30 pM in the North Atlantic ocean (Panzeca et al., 2008), and from undetectable to 500 pM in the Southern California-Baja California coast (Sañudo-Wilhelmy et al., 2012). Vitamin B_6_ concentrations in the WTNA ranged from undetectable to 40 pM and were generally lower than previous measurements from the North Pacific Ocean, specifically from the Santa Monica Basin (70–284 pM), Rosario (255–386 pM), and Magdalena (40–393 pM) from the upper 150 m, and Vizcaino (159–360 pM) from the upper 120 m of the water column (Sañudo-Wilhelmy et al., 2012). However, they fell within the range measured at Soledad (3.5–49 pM) from the upper 150 m and Pescadero (4.6–180 pM) from the upper 180 m of the water column (Sañudo-Wilhelmy et al., 2012). In summary, the concentrations of B-vitamins observed in this study were consistent with previous results showing they vary spatially, and are often found below the limit of detection.

**Table 4:** Global B vitamin concentrations including current and previous studies. n/a not available, n/d not detectable.

Location	Lat.	Long	Depth (m)	Method	Range (pM)	Reference
**B**_**1**_
North Atlantic Ocean	4.3–12.4	−56.8 to −45	150	direct	0.05–1000	This study
North Pacific Ocean	58	134 to 137	150	bioassay	33–1633	Natarajan and Dugdale (1966)
Subarctic Pacific Ocean	52–58	153 to 170	150	bioassay	36–1500	Natarajan (1970)
Southern CA Coastal zone	−117 to −119	32 to 34	80	bioassay	16–133	Eppley et al. (1972)
Stony Brook Harbor	n/a	n/a	surface	direct	230–310	Okabamichael and Sañudo-Wilhelmy (2005)
North Atlantic ocean	45–66	−14 to −24	surface	direct	0.7–30	Panzeca et al. (2008)
Southern CA-Baja	113–119	34–23	180	direct	0.34–122	Sañudo-Wilhelmy et al. (2012)
**B**_**6**_						
North Atlantic Ocean	4.3–12.4	−56.8 to −45	150	direct	n/d to 40	This study
Santa Monica Basin	−119.03	33.84	150	direct	70–284	Sañudo-Wilhelmy et al. (2012)
Rosario	−116.08	29.8	150	direct	255–368	Sañudo-Wilhelmy et al. (2012)
Vizcaino	−114.52	27.01	120	direct	159–360	Sañudo-Wilhelmy et al. (2012)
Soledad	−112.71	25.22	150	direct	3.5–49	Sañudo-Wilhelmy et al. (2012)
Magdalena	−111.57	23.2	150	direct	40–393	Sañudo-Wilhelmy et al. (2012)
Pescadero	−108.2	24.28	180	direct	4.6–180	Sañudo-Wilhelmy et al. (2012)

Recent studies on the role that B-vitamins play in marine ecosystems have shown that they can limit or co-limit primary production (Panzeca et al., 2006,^2008^; Bertrand et al., 2007; Gobler et al., 2007; Tang et al., 2010). Although this study did not directly investigate the effects of vitamin additions on biological processes, some conclusions can be drawn from the correlations between vitamin concentrations and rates of N_2_ and C fixation. This study found a significant increase in C fixation with increasing ambient B_1_ concentrations at low-salinity station 9.1 in both size classes (*p* value í0.004, **Table 2**). At low-salinity station 4, the lack of correlation between C fixation and B_1_ concentrations could be explained by the high abundance of the diatom *Coscinodiscus* sp. Based on isolates that have been studied it appears that this diatom species does not require vitamin B_1_ (Croft et al., 2006) and likely contributed to the majority of C fixation at this station. Significant increases in N_2_ fixation were also found with increasing B_1_ in the large size class at station 1 (*p* value 0.029) and oceanic station 7 in the small size class (*p* value <0.045). These data suggest that B_1_ may be limiting or co-limiting N_2_ fixation in some areas of the WTNA since low PO_4_^3-^ concentrations were also measured at station 7, and PO_4_^3-^ has been previously shown to limit N_2_ fixation (Sañudo-Wilhelmy et al., 2001; Mills et al., 2004; Webb et al., 2007; Moutin et al., 2008; Van Mooy et al., 2009). Hence, vitamin B_1_ appears to be playing a role in C and N_2_ fixation in both riverine influenced and open ocean stations. These results are consistent with the role of B_1_ in C metabolism but the role B_1_ plays in N metabolism is less clear. However, pyruvate-ferredoxin oxidoreductase, an enzyme crucial for electron transfer to nitrogenase, requires thiamin (Brostedt and Nordlund, 1991; Bothe et al., 2010) and some diazotrophs have been shown to be B_1_ auxotrophs, suggesting that B_1_ availability in the environment may be limiting the N biogeochemical cycle. N_2_ fixation was found to increase with increasing vitamin B_1_ at one low-salinity station; however, this was not observed at other stations. Therefore, further investigations such as vitamin addition experiments which show an increase of N_2_ fixation with B_1_ amendments, are required to fully understand the role of this vitamin in the WTNA N and C cycles. However, the tight correlation between B_1_ and C fixation observed at station 9.1 (**Figure 3D**) suggests that this vitamin may also be important for C fixation in the WTNA, and argues for further study.

N_2_ fixation co-varied with vitamin B_6_ at low-salinity station 9.1; significant positive relationships were found in larger size class between vitamin B_6_ and N_2_ fixation (*p* value < 0.017). However, there was not a significant relationship between N_2_ fixation and concentrations of vitamin B_6_ at the other stations. No significant relationships were observed between C fixation and vitamin B_6_ concentrations at any stations. Independence of vitamin B_6_ and N_2_ fixation can be explained by other factors; for instance, at station 7, low nutrient concentrations were observed and dissolved P may have limited N_2_ fixation, while station 4 was dominated with the diatom *Coscinodiscus *sp. whose requirements for B_6_ are currently unknown. Thus, at some stations N_2_ fixation appears dependent on B-vitamins, which appears to be limiting or co-limiting biogeochemical cycles in the WTNA. Since there were few correlations between vitamin concentration and rate measurements, either standing concentrations are a poor measure, auxotrophic phytoplankton are not commonly abundant, or they are getting their vitamins through symbiosis (Croft et al., 2005). However, to determine the extent that N and C cycles are actually dependent on vitamin B_6_, more extensive studies including vitamin addition experiments will be required.

Multiple linear regression models were used to identify the environmental variables that correlated with biogeochemical cycles in the WTNA Ocean during our study. Variables correlating to C fixation in the small size class included Si, vitamin B_1_, and temperature. Two of these variables, Si and water temperature, were also identified as factors affecting the distribution of N_2_ and C fixing organisms in previous studies (Coles and Hood, 2007; Foster et al., 2007; Webb et al., 2007; Sohm and Capone, 2008; Hynes et al., 2009; Van Mooy et al., 2009; Sohm et al., 2011a,2011b). Model results were consistent with the role that vitamin B_1_ plays in the Calvin cycle and C metabolism (Natarajan, 1970; Jordan, 2003). Our analysis showed that temperature, MLD, and vitamin B_6_ correlated to N_2_ fixation in the greater size fraction. Measured N_2_ fixation rates were on average an order of magnitude less than modeled rates except at depths where the highest rates of N_2_ fixation were measured. When the highest rates of N_2_ fixation were observed, measured rates were an order of magnitude greater than the modeled rates (**Figure 4**). This pattern was observed across all station types and resulted in the models low *R*^2^ value. However, this is consistent with the role vitamin B_6_ plays in catalyzing many diverse amino acid transformations (Percudani and Peracchi, 2009), specifically with the assimilation of NH_3_ into the amino acids glutamine and glutamate. Collectively, these results suggest that vitamin B_1_ and B_6_ could be important organic growth factors affecting biologically mediated C and N_2_ fixation in the WTNA Ocean.

Insights into the potential ecological importance of B-vitamins have been investigated by determining half-saturation constants (*K*_s_) for maximal growth for vitamins B_1_ and B_12_ for some phytoplankton species (Tang et al., 2010). However, the *K*_s_ for diazotrophic microorganisms and B-vitamins have yet to be determined. The *K*_s_ of maximal growth rates for different phytoplankton species for vitamin B_1_ ranged from 6 to 184 pM. Some of our measured concentrations of B_1_ were below the *K*_s_ suggesting that vitamin B_1_ may be a limiting growth factor in the WTNA. Future studies are needed to determine the *K*_s_ for maximal growth on different B-vitamins of endemic WTNA plankton species, which will help to establish the ecological framework and importance of directly measured environmental B-vitamin concentrations.

The influence of the Amazon River plume on B-vitamin concentrations and the sources of B-vitamins in the WTNA are still unresolved. Although it has been hypothesized that fresh water inputs from rivers and groundwater can be a source of B-vitamins to marine systems (Gobler et al., 2007), clear patterns were not observed to support this in the WTNA Ocean. As a general trend, there was an increase in B-vitamins as salinity decreased but no linear relationship was observed, suggesting that mixing of river and seawater did not solely control it. An inverse correlation was observed with vitamin B_1_ concentration and SSS (*R* value 0.25, data not shown), but no correlation was observed between vitamin B_6_ and SSS (*R* value 0.002, data not shown). The surface water sampled during this cruise was estimated to be nearly 30 days out from the mouth of the river, and may explain the weak correlations found between SSS and B-vitamin concentrations. Further studies investigating B vitamin concentrations near the discharge point of the Amazon River should help resolve whether the river is a source of vitamins to the WTNA. In addition, the removal processes of B-vitamins are poorly understood, and the half-life of these vitamins has yet to be determined. However, the half-life of some vitamins (B_1_ and B_12_) in seawater has been shown to occur on time scales from days to weeks (Gold et al., 1966; Carlucci et al., 1969), suggesting that they are highly dynamic and that local production may be an important biologically available source of B-vitamins. Our understanding of the ecological importance of B-vitamins in marine systems is continuing to increase, with the current study demonstrating that B-vitamins are highly variable and could significantly influence both N_2_ and C fixation in the WTNA Ocean. However, further studies are needed to determine the sources, sinks, and cycling of B-vitamins in oceanographic sensitive marine systems, such as the WTNA.

## Conflict of Interest Statement

The authors declare that the research was conducted in the absence of any commercial or financial relationships that could be construed as a potential conflict of interest.
